# A neuroprosthesis for tremor management through the control of muscle co-contraction

**DOI:** 10.1186/1743-0003-10-36

**Published:** 2013-04-15

**Authors:** Juan Álvaro Gallego, Eduardo Rocon, Juan Manuel Belda-Lois, José Luis Pons

**Affiliations:** 1 Bioengineering Group, Consejo Superior de Investigaciones Científicas (CSIC), , Ctra Campo Real km 0.2 - La Poveda, 28500 Arganda del Rey, Spain; 2 Instituto de Biomecánica de Valencia, Universitat Politècnica de València, Camino de Vera s/n ed. 9C, E46022 Valencia, Spain; 3 Grupo de Tecnología Sanitaria del IBV, CIBER de Bioingeniería, Biomateriales y Nanomedicina (CIBER-BBN) Valencia, Spain

**Keywords:** Tremor, Neuroprosthesis, Neurostimulation, Parkinson’s disease, Essential tremor, Adaptive filtering, Functional electrical stimulation, Human-Machine Interface

## Abstract

**Background:**

Pathological tremor is the most prevalent movement disorder. Current treatments do not attain a significant tremor reduction in a large proportion of patients, which makes tremor a major cause of loss of quality of life. For instance, according to some estimates, 65% of those suffering from upper limb tremor report serious difficulties during daily living. Therefore, novel forms for tremor management are required. Since muscles intrinsically behave as a low pass filter, and tremor frequency is above that of volitional movements, the authors envisioned the exploitation of these properties as a means of developing a novel treatment alternative. This treatment would rely on muscle co-contraction for tremor management, similarly to the strategy employed by the intact central nervous system to stabilize a limb during certain tasks.

**Methods:**

We implemented a neuroprosthesis that regulated the level of muscle co-contraction by injecting current at a pair of antagonists through transcutaneous neurostimulation. Co-contraction was adapted to the instantaneous parameters of tremor, which were estimated from the raw recordings of a pair of solid state gyroscopes with a purposely designed adaptive algorithm. For the experimental validation, we enrolled six patients suffering from parkinsonian or essential tremor of different severity, and evaluated the effect of the neuroprosthesis during standard tasks employed for neurological examination.

**Results:**

The neuroprosthesis attained significant attenuation of tremor (*p*<0.001), and reduced its amplitude up to a 52.33±25.48*%*. Furthermore, it alleviated both essential and parkinsonian tremor in spite of their different etiology and symptomatology. Tremor severity was not a limiting factor on the performance of the neuroprosthesis, although there was a subtle trend towards larger attenuation of more severe tremors. Tremor frequency was not altered during neurostimulation, as expected from the central origin of Parkinson’s disease and essential tremor. All patients showed a good tolerance to neurostimulation in terms of comfort and absence of pain, and some spontaneously reported that they felt that tremor was reduced when the neuroprosthesis was activated.

**Conclusions:**

The results presented herein demonstrate that the neuroprosthesis provides systematic attenuation of the two major types of tremor, irrespectively from their severity. This study sets the basis for the validation of the neuroprosthesis as an alternative, non-invasive means for tremor management.

## Background

Pathological tremors, understood as the ensemble of tremors that are cause of functional disability, constitute the most prevalent movement disorder. Although obtaining an estimate of its prevalence is challenging [1], a recent population study showed that tremor affects 15% of those with age ranging between 50 and 89 years [2]. According to some estimates, more than 65% of those who suffer from pathological tremor—referred to as tremor in the remainder of the paper—at the upper limb report serious difficulties when performing their activities of daily living (ADLs) [3]. Therefore, tremor has an enormous impact on the independence and quality of life of many.

The two most relevant conditions, in terms of prevalence, that cause tremor are essential tremor (ET) and Parkinson’s disease (PD). ET is believed to originate by abnormal neuronal oscillations at the cerebellar and thalamocortical pathways [4,5] and, according to some, constitutes the most common movement disorder in adults [1] (it affects ∼ 5% of people over age 65 [6]). Much is yet unknown about ET, for example there is still no consensus on whether it is a neurodegenerative or non-degenerative disease [7]. PD is a neurodegenerative disease that arises from the death of dopaminergic nigrostriatal neurons, causing abnormal oscillations in the loop linking the cortex, basal ganglia and thalamus [8]. PD is estimated to afflict more than ten million people all over the world [9]. In addition to them, up to eight more syndromes are acknowledged to cause tremor [10]. Importantly, no tremor is yet fully understood [4], which hampers the development of novel therapies, and the refinement of the existing ones.

Tremors are managed either through pharmacotherapy or neurosurgery, the latter consisting in the implantation of a deep brain stimulator (DBS), or the realization of therapeutical lesions in deep brain structures (gamma knife thalamotomy). Nonetheless, both pharmacotherapy and neurosurgery carry drawbacks associated. Drugs often induce side effects [11,12], and show decreased effectiveness over years of use [13,14], while DBS is related to increased risk of intracranial haemorrhage [15] and psychiatric manifestations [16], and the percentage of eligible patients is extremely low [17]. As for the tremors themselves, the mechanisms accounting for the alleviation of their symptoms by these therapies are not fully elucidated.

Among the alternative approaches for tremor management, the application of mechanical loads, either forces or masses, is regarded as one of the most promising solutions. A number of works show how adequate inertial (mass) loading of the tremulous limb reduces the amplitude of most types of tremor [18,19], and so does the application of forces with certain characteristics. Force loads typically consist of viscous fields [20,21], although the effect of added stiffness [22] and inertia [23] on tremor has also been investigated. Furthermore, voluntary [24,25] or artificially elicited [26] muscle contractions affect the severity of the tremor, by generating forces on the musculoskeletal system.

These findings motivated the development of orthotic devices for tremor attenuation, being the majority of them wheel-chair mounted [27,28] or fixed to an external frame [29,30]. To the best of our knowledge, the only ambulatory system for tremor attenuation through mechanical loading extensively validated with patients is the wearable robot WOTAS [21]. This system attained consistent attenuation of tremors over a certain severity but, however, did not fulfill users’ expectations in terms of cosmetics and aesthetics [21]. Besides, it was found that the intrinsic difficulty of transmitting external forces to the skeletal system through the soft tissues hampered the improvement of solutions of this kind. Therefore, here we present a novel approach for tremor management aimed at circumventing these limitations. Our novel solution consists of a neuroprosthesis (NP) that applies forces to the tremulous limb through transcutaneous neurostimulation. We show that by co-contracting the affected muscles it is possible to alter the inherent low pass filter properties of human muscles [31] and attenuate the tremor, without affecting the concomitant voluntary movement. Our results demonstrate that the NP provides systematic alleviation of tremors of different etiology and characteristics, avoiding discomfort and pain. Notice that transcutaneous neurostimulation for tremor management had been preliminarily evaluated in [32]. There, an alternanting stimulation pattern that opposed the tremor activation bursts was employed to reduce its severity [26]. The system was, however, table mounted.

This paper is organized as follows. First, we describe the NP and the controller it implements, together with the experimental protocol and methods employed for its validation. Afterwards, we review and discuss the results achieved. The manuscript ends up with conclusions summarizing the outcomes of the study.

## Methods

### The neuroprosthesis for tremor management

**Figure 1:**
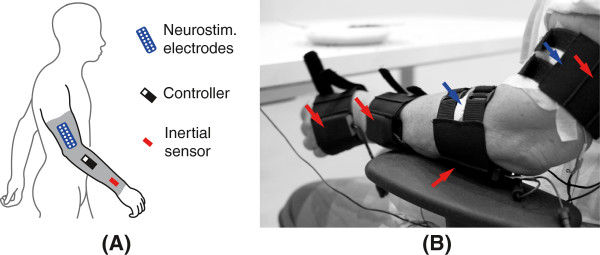
**NP for tremor management.** The concept design (**A**) envisioned the NP as a textile substrate that integrated the neurostimulation electrodes, the gyroscopes and the control electronics. The final prototype (**B**) consisted of two pairs of textile supports that incorporated the solid gyroscopes, and off the shelf transcutaneous electrodes. This permitted personalizing the prototype to each patient. In (B), red arrows point at the gyroscopes (placed in the textile supports), and blue arrows at the neurostimulation electrodes (the color code corresponds to that employed for the NP constituents in (A)). The electrodes over the flexor carpi ulnaris and the biceps brachii are not visible in the picture.

The NP here presented employed transcutaneous neurostimulation to apply mechanical loads as a means of alleviating upper limb tremor. Loads were applied concurrently at a pair of antagonist muscles—in co-contraction—in such a way that joint impedance was adequately manipulated. The elicited co-contraction level was continuously adapted to the ongoing severity of the tremor, which was estimated from the recordings of solid state gyroscopes. Figure 1(A) shows the concept design we followed. The NP actuated at the wrist and elbow joints, because they have the largest impact on disability [33], and took the shape of a textile substrate that integrated the neurostimulation electrodes and the sensors that drove the system. The textile substrate could be worn underneath the clothes, thus satisfying users’ expectations in terms of cosmetics and usability. Figure 1(B) shows a patient wearing the final prototype, which had modular design (it was not a continuous garment as represented in the concept) in order to maximize its adaptability to user’s anatomy.

The rationale behind the use of co-contraction was to increase simultaneously the stiffness and viscosity of the tremulous limb, which in turn would decrease the cut-off frequency (∼ 2–3 Hz [31,32,34]) of the inherent low pass filter response of muscles, filtering out the tremor. Indeed, co-contraction is exploited by the intact central nervous system to stabilize the limbs during specific tasks [35,36]. Furthermore, this could be implemented directly by increasing muscle activation, because stiffness and viscosity are monotonic functions of the contraction level [37].

A simplified explanation of our approach is given next. Equation (1) represents a human joint with the NP attached to it. There, the human joint was modeled as a second order linear time invariant system (as done in many works in the literature, e.g. [30,31,38]), while the NP was represented as a variable stiffness and viscosity, according to what was explained before. Given that the NP behaves as a system acting in parallel to the limb, the resultant response could be modeled as [30,38]:

(1) $$\frac{\theta (s)}{T(s)}=\frac{G}{Is^{2}+(D+D_{\text{NP}})s+(K+K_{\text{NP}})}$$

where *θ*(*s*) is the tremulous component of movement (estimated as described in epigraph ‘Tremor Parameterization’), *T*(*s*) represents the torque that generates the tremor, *I*, *D*, and *K* stand for the inertia, viscosity and stiffness of the joint respectively, and *G* is the magnitude of the response of the resultant system (that is function of its mechanical parameters). The viscosity and stiffness added by the NP as a result of increased muscle contraction are denoted by *D*_NP_ and *K*_NP_, and satisfy *D*_NP_,*K*_NP_≥0, being *D*_NP_,*K*_NP_=0 if the NP was not activated.

**Figure 2:**
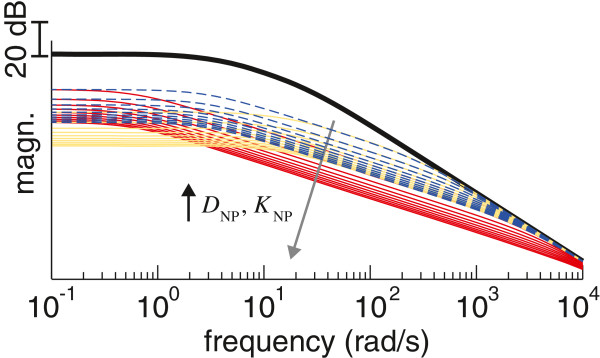
**Rationale behind the tremor suppression approach.** Frequency response of a human joint, showing how it is modified by co-contraction of the antagonist muscle pair. The data for the human joint without the neuroprosthesis (black trace) corresponds to the model given in (1) fitted to the parameters identified for the wrist in [39]. The remainder traces represent how the response of the joint is modified by a concurrent increase in viscosity and stiffness (defined as a multiple of the parameters *D* and *K*), and correspond to: **i**) equal increments of *D*_NP_ and *K*_NP_ (in multiples of 10, from 10 to 100, shown as blue traces), **ii**) larger increments of *D*_NP_ (in multiples of 50, from 50 to 500) than of *K*_NP_ (in multiples of 10, from 10 to 100, shown as red traces), and **iii**) larger increments of *K*_NP_ (in multiples of 50, from 50 to 500) than of *D*_NP_ (in multiples of 10, from 10 to 100, shown as yellow traces). For all cases, larger values of *D*_NP_ and *K*_NP_ cause the response of the joint to have smaller magnitude (as indicated by the arrow in gray).

Given that the analytical expression that relates the changes in *D*_NP_ and *K*_NP_ caused by the NP to the cut off frequency of the resultant system (represented in (1)) is very complex, in Figure 2 we show how a concurrent increase *D*_NP_ and *K*_NP_ would affect the frequency response of the joint. There, it is displayed how the cut off frequency would be decreased in a nonlinear manner when the contraction level, and thus the resultant stiffness, *K*+*K*_NP_, and viscosity, *D*+*D*_NP_, of the joint [37], were increased. The change in the magnitude of the response observed in Figure 2 illustrates how co-contraction stabilizes the limb, as reported in the literature [35,36].

In our case, the level of artificially elicited co-contraction was adapted to the characteristics—instantaneous amplitude and frequency—of the tremor. An independent controller was implemented for each joint, because the characteristics of tremor differs among them, and show a non-stationary behavior that is normally uncoupled. Neurostimulation was controlled as follows. Tremor frequency defined when the control output was to be updated, while tremor amplitude modulated the amount of current to be injected to each muscle. Tremor parameters estimated during a (tremor) period were employed to generate the control action in the subsequent one, analogously to repetitive control theory [40]. This provided the controller with a certain predictive nature. The reason to modulate the level of neurostimulation independently for each muscle in the pair of antagonists was twofold: first, because of their different electrophysiological response to neurostimulation, and second, because this response also varies with time in a different manner for each muscle. Neurostimulation was modulated in amplitude; current frequency and pulse width were kept constant. Details on the adaptive filter employed to estimate instantaneous tremor amplitude and frequency, and on the control algorithm, are given next.

#### Tremor parameterization

Joint rotation was simply obtained by computing the difference of a pair of solid state gyroscopes, which were located at the distal and proximal segments following [41]. Next, given that the concomitant voluntary and tremulous movements are additive [42], the total joint rotation *y*(*k*) measured at each joint was expressed as:

(2) $$y(k)=y_{\text{tremor}}(k)+y_{\text{vol}}(k)$$

where *y*_*tremor*_(*k*) represents the tremor, and *y*_*vol*_(*k*) the component of voluntary movement. A two–stage filter [42], summarized next, separated the voluntary movement and the tremor in order to estimate the instantaneous parameters of the latter. This information was in turn employed by the controller to drive the NP.

The estimation of voluntary movement relied on the fact that tremors occur at a higher frequency (in the 3–12 Hz band [10]) than that employed to perform the ADL (below 2 Hz [43]). Therefore, concomitant tremulous and voluntary components of movement were separated (in the first stage) based solely on their different frequency bands. This was implemented with a *g*−*h* filter [44] that estimated the voluntary component of motion. The *g*−*h* filter is an adaptive algorithm that incorporates a first order model of voluntary movement, and is formulated as follows:

(3) $$\begin{array}{ll} & x_{k,k}=x_{k,k-1}+g_{k}(y_{k}-x_{k,k-1})\; \end{array}$$

(4) $$\begin{array}{ll} & {\dot{x}}_{k,k}={\dot{x}}_{k,k-1}+\frac{h_{k}}{T_{s}}(y_{k}-x_{k,k-1})\; \end{array}$$

(5) $$\begin{array}{ll} & x_{k+1,k}=x_{k,k}+T_{s}\;{\dot{x}}_{k,k}\; \end{array}$$

(6) $$\begin{array}{ll} & {\dot{x}}_{k+1,k}={\dot{x}}_{k,k}\; \end{array}$$

where *x*_*k*,*k*_ and ${\dot{x}}_{k,k}$ represent the estimation of voluntary movement and its derivative, which were computed in (3) and (4) by updating the previous predictions *x*_*k*,*k*−1_ and ${\dot{x}}_{k,k-1}$ with the current measurement *y*_*k*_. Equations (5) and (6) predicted the future value of the voluntary movement and its derivative, *x*_*k*+1,*k*_ and ${\dot{x}}_{k+1,k}$, based on the first order model included in the filter; *T*_*s*_ represents the sampling period. Filter parameters *g*_*k*_ and *h*_*k*_ were considered to be constant, and chosen so that they fulfill the next relationship, which minimizes the squared error of previous measurements assigning less significance to older values [44]:

(7) $$\begin{array}{lll} & g=1-\theta^{2}\;\\ & h={\left(1-\theta \right)}^{2}\; & \; \end{array}$$

By selecting an adequate value of parameter *θ* we obtained a precise estimation of voluntary movement with negligible delay, which is of foremost importance to get an accurate tracking of tremor parameters. The delay introduced by Butterworth, Chebyshev and elliptic low pass filters was notably higher, which motivated our choice.

Instantaneous tremor frequency and amplitude were subsequently estimated (in the second stage) with a Weighted Frequency Fourier Linear Combiner (WFLC) [45] and a Kalman Filter [46] in the following manner: the WFLC tracked tremor frequency, and this value was fed into the Kalman filter, which estimated the instantaneous amplitude of tremor. This cascade architecture permited optimizing the tracking of both parameters [45].

The WFLC adapts concurrently the amplitude terms and fundamental frequency of a truncated Fourier series based on the Least Mean Squares recursion [45]. Our Kalman filter also implemented a first order harmonic representation of the tremor, and was formulated as follows:

(8) $$\begin{array}{ll} \;\left[\begin{array}{c} A_{k,k-1}\\ B_{k,k-1}\\ tr_{k,k-1} \end{array}\right]\;= & \left[\begin{array}{ccc} 1 & 0 & 0\\ 0 & 1 & 0\\ \cos(\underset{t}{\sum }\omega_{t}) & \sin(\underset{t}{\sum }\omega_{t}) & 0 \end{array}\right]\;\left[\begin{array}{c} A_{k-1,k-1}\\ B_{k-1,k-1}\\ tr_{k-1,k-1} \end{array}\right]\; \end{array}$$

(9) $$\begin{array}{ll} & \;y_{\text{tremor},k}=tr_{k,k-1}\; \end{array}$$

where *A* and *B* are the amplitude terms of the Fourier series, and *ω*_*t*_ is the instantaneous frequency derived with the WFLC; *tr*_*k*,*k*−1_ represents the estimation of tremor based on these three parameters. Measurement noise covariance was defined as $R(k)=\sigma_{\tau }^{2}$, whereas process noise **Q**(*k*) was modeled as a diagonal matrix, assuming independence of the state variables. The measurement noise covariance was related to the accuracy of the estimation of *y*_*tremor*,*k*_ with the *g*−*h* filter, while the process noise covariance determined the adaptation rate of the amplitude terms of the truncated Fourier series, *A* and *B*.

#### Control algorithm

The controller that regulated neurostimulation amplitude was a rule-based proportional-integral law (10), where the integral gain was switched between two values depending on the amplitude of the residual tremor (11). By switching the value of *K*_*i*_(*tr*_*k*,*k*−1_) to 0, the controller neglected the integral term when the amplitude of the tremor was small, i.e. below the threshold *th*_*intgain*_, which avoided possible unnecessary periods of stimulation when the tremor was very mild and did not pose a functional problem. Furthermore, the integral was reset when tremor amplitude decreased below a certain threshold, *th*_*intreset*_. This avoided adding the residual tremor to the integrator when tremor amplitude was very small, which prevented brisk transients if the tremor developed again. The inclusion of a integral term was very important to compensate for the time varying response to neurostimulation that characterizes this type of applications, which manifests overall in terms of muscle fatigue and accommodation to neurostimulation [47]. The controller was defined as:

(10) $$\begin{array}{ll} u_{k}=K_{p}tr_{k,k-1}+K_{i}(tr_{k,k-1})\overset{k}{\underset{j=1}{\sum }}tr_{j}T_{s} & \; \end{array}$$

(11) $$\begin{array}{ll} K_{i}(tr_{k,k-1})=\left\{\begin{array}{cc} K_{i} & \;\text{if}\;tr_{k,k-1}\geq th_{\text{int}\;\text{gain}}\\ 0 & \;\text{if}\;tr_{k,k-1}<th_{\text{int}\;\text{gain}} \end{array}\right) & \; \end{array}$$

where *K*_*p*_ and *K*_*i*_(*tr*_*k*,*k*−1_), are the controller gains (the value of the latter was switched depending on tremor amplitude, as shown in (11)), and *u*(*k*) the control action. Importantly, a saturation was applied to *u*(*k*) in order to limit the electrical charge injected to the muscle. This value was defined for each muscle during a calibration phase.

The NP was triggered when the onset of tremor and its initial frequency were detected from the solid state gyroscopes. Tremor onset was immediately obtained by comparing the estimated tremor amplitude, *tr*_*k*,*k*−1_, to a threshold, *tr*_*th*_. A gross estimation of tremor frequency was computed as the maximum of the amplitude spectrum in the 3–12 Hz band [10]. This estimation was used to initialize the WFLC. In order to double-check tremor detection, we used the following criterion that relied on the spectral characteristics of the total movement: if the ratio of tremor to voluntary movement (*TVR*) was larger than a certain value defined manually (*TVR*_*th*_), we considered that the spectrum reflected the presence of tremor. In case this condition was not fulfilled, the next window of equal length was employed for the calculation; 50% overlapping was allowed. The *TVR* was defined as:

(12) $$\text{TVR}=\frac{\overset{f=12}{\underset{f=3}{\sum }}X_{f}}{\overset{f=3}{\underset{f=0}{\sum }}X_{f}}$$

where $\overset{f=3}{\underset{f=0}{\sum }}X_{f}$ and $\overset{f=12}{\underset{f=3}{\sum }}X_{f}$ represent the integral of the amplitude spectrum in the 0–3 Hz and 3–12 Hz bands. Similar ratios have been employed in other works about tremor, e.g. for EMG analysis [48].

### Patients, apparatus and experimental protocol

We present results obtained by evaluating the NP in 6 patients (1 female) suffering from either PD (*n* = 2) or ET (*n* = 4). Average age was 68.2±13.8 years. Tremor amplitude, according to neurological rating, ranged from very mild to severe (from 3 to 30 according to Fahn-Tolosa-Marin score^a^[49]). ET patients were asked to interrupt their intake of medication 24 h before the recordings, while PD patients continued the use of antiparkinsonian medication. All patients signed a written informed consent to participate. The protocol was approved by the Polytechnic University of Valencia, which warranted its accordance with the Declaration of Helsinki. Details on patients are given in Table 1.

**Table 1:** Details of patients

**Patient**	**Disorder**	**Gender**	**Age**	**Tremor frequency (Hz)**	**Tremor severity**
01	ET	M	69	∼ 4.2	27
02	PD	M	44	∼3.5	30
03	ET	M	82	∼ 5.1	n.a. (moderate)
04	PD	M	67	∼ 4.8	3
05	ET	F	66	∼ 4.5	17
06	ET	M	81	∼ 8.0	n.a. (mild)

Patients wore the NP at the most affected side and were asked to perform the clinical task that made their tremor more evident. Typical exercises for neurological examination were considered: resting the arm on the lap, keeping both arms outstretched, the finger to finger test, and the finger to nose test [10]. Patients were sitting in a comfortable armchair during the whole session. The NP recorded both wrist flexion/extension and elbow flexion/extension with two pairs of solid state gyroscopes (Technaid S.L., Madrid, Spain), although we present results only for the wrist, because it was where the tremor was visible for all patients. Neurostimulation was delivered at the flexor carpi ulnaris and extensor carpi radialis with a multichannel monopolar neurostimulator with charge compensated pulses (UNA Systems, Belgrade, Serbia); the common electrode was located at either the dorsal or volar side of the wrist. Maximum neurostimulation amplitude for each muscle was personalized during an initial calibration phase; pulse width and frequency were set to either 300 *μ*s and 40 pps or 250 *μ*s and 30 pps respectively, depending on the patient: we used the first combination of values in patients that needed high current density to elicit a visible muscle contraction. The controller was implemented in a stand alone computer (QNX Software Systems, Ontario, Canada). Figure 1(B) shows a patient wearing the NP.

Each patient performed a number of repetitions of two types of trials; the duration of each was 30 s. The first type of trial consisted of two 15 s sub-periods, during the second of which the NP was activated. In the second type of trial, the NP was never activated. This type of trial was included in the protocol to avoid a possible distortion of the study due to, for example, a natural reduction of tremor amplitude over time. Both types were randomized, and the experimental design was balanced using an optimal algorithm, in which the repetitions were ordered by concatenating latin squares [50]. In total, each patient performed between 6 and 12 repetitions. Patients with mild or moderate tremor were asked to count mentally backwards during the experiments to exacerbate their tremor [51,52], and the two with PD counted out loud at the beginning of the session as recommended in [52]. Patients with severe or very persistent tremor were not asked to count during the recordings to avoid excessive mental stress, which could have possible ethical implications.

### Data analysis

To quantify the effect of the NP on tremor amplitude, we computed the ratio *R*_*att*_ of the integral of the power spectral density of the tremor during the part of the trial with co-contraction, to the same variable without it [21]. Before, data were split into 1 s non-overlapping windows to minimize the effects of eventual non-stationarities, and zero padded; *R*_*att*_ was calculated with the median values for both conditions. We computed *R*_*att*_ also for the trials during which the NP was not activated, in order to investigate whether it had a real effect on the tremor. To this end, we considered equivalent periods for its calculation.

We assessed whether the NP had a real effect on tremor amplitude by comparing *R*_*att*_ in the ensemble of trials of the two types (pooled trials in which these NP was activated or not) with a Mann-Whitney test, given that the pooled datasets did not conform normality after transformation (Kolmogorov-Smirnov test, *p*<0.05). We also evaluated whether there was an effect on tremor frequency with a Mann-Whitney test. Throughout the paper, values are reported as mean ± SD.

The parameters for the tremor parameterization algorithms and the controller were set to: i) for the *g*−*h* filter, *θ*= 0.900, ii) for the Kalman filter, $\sigma_{\tau }^{2}=$ 0.01, **Q**(*k*)=*diag*(1,1,0), and iii) for the WFLC, *μ*_0_= 0.001, *μ*_1_= 0.01, *μ*_*b*_= 0.01, *M*= 1, *f*_0_ was initialized to the value computed from the amplitude spectrum at tremor onset (data epoch of 2 s with zero padding). The parameters of these three algorithms were obtained by analyzing offline a previously recorded dataset. Controller gains were selected manually based on the amplitude of the tremor that was observed during calibration and the required neurostimulation amplitude. During this phase, we also defined the saturation level (maximum current amplitude) the controller could deliver at each muscle. Thresholds for tremor onset, frequency estimation, integral reset, and gain switching were *tr*_*th*_= 0.1 rad/s, *TVR*_*th*_= 3, *th*_*intreset*_= 0.1 rad/s, and *th*_*intgain*_= 0.1 rad/s.

## Results and discusison

### Results

**Figure 3:**
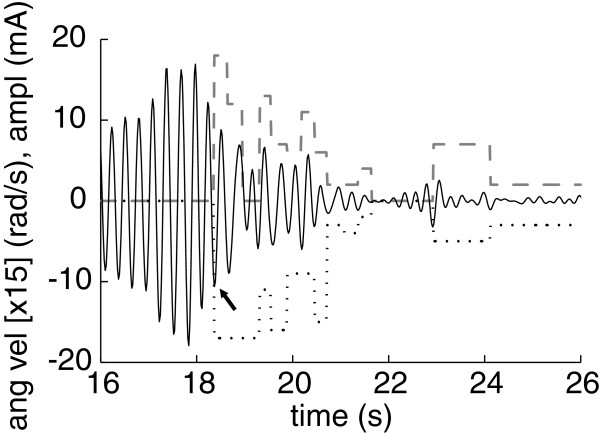
**Example of the controller of the NP.** The plot shows the estimation of tremor (solid line) and the amplitude of the current applied at the extensors (dashed line) and flexors (dotted line). The instant at which the NP was triggered is signaled with an arrow. Angular velocity is scaled [x 15] for visualization sake; a positive value corresponds to wrist extension.

**Figure 4:**
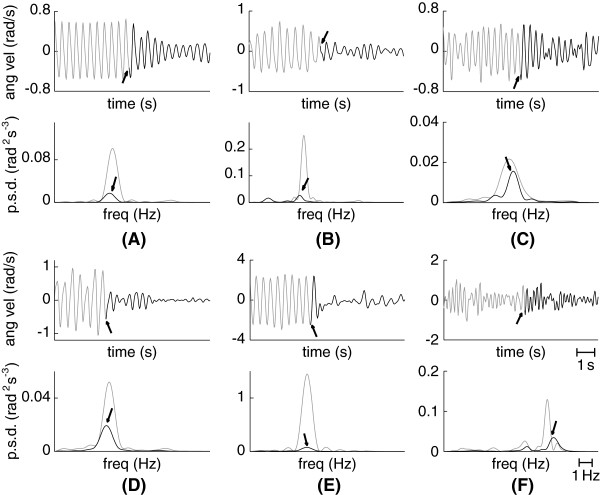
**Examples of reduction of tremor amplitude with the NP.** Each subplot in this graphic shows a representative trial for each patient, and compares the amplitude of the tremor before (gray) and after (black) the NP was activated. Top plots represent the data in the time domain, whereas bottom plots represent the same data in the frequency domain (we show the mean power spectral density for the neurostimulation and non-neurostimulation periods). In the time domain, the moments at which the NP was triggered are signaled with an arrow; in the frequency domain, the arrow points at the tremor peak while the NP was activated. Examples correspond to: patient 01 (**A**), patient 02 (**B**), patient 03 (**C**), patient 04 (**D**), patient 05 (**E**) and patient 06 (**F**).

Figure 3 shows a typical example of the performance of the NP, representing the current amplitude delivered at each muscle together with the ongoing tremor (estimated with the two-stage algorithm presented in epigraph ’Tremor Paramenterization’). We observe that ∼ 2.5 s after the system was triggered, the amplitude of the tremor was drastically reduced, and the control action diminished and became 0. In the case of this patient, low current kept the limb considerably stabilized, i.e. prevented the tremor from reappearing with the same amplitude that he normally exhibited. The importance of the integral term is appreciated at time ∼ 19 s at the flexors, when the controller maintained the maximum (saturation) current amplitude to compensate for the still severe tremor. In Figure 4 we show a representative example of tremor alleviation with the NP for each patient, in the time and frequency domains. They range from very large attenuation (see Figure 4(B) and 4(E)) to mild reduction of tremor amplitude (see Figure 4(C)). Additional file 1 provides two video examples of the performance of the NP.

**Figure 5:**
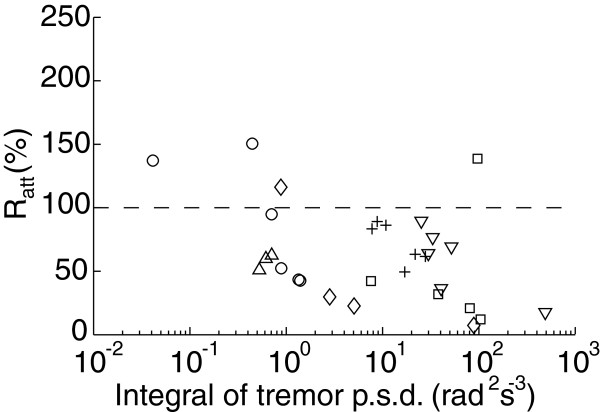
**Summary of all the trials in which the NP was active.** The plot shows tremor attenuation computed with *R*_*att*_ as a function of tremor severity. Each marker stands for a single trial. The plot is interpreted as the further below the dashed line (geometrical locus of *R*_*att*_ = 100%), the larger the attenuation. Patients are codified as follows: ▿ corresponds to patient 01, □ to patient 02, + to patient 03, ∘ to patient 04, â™¢ to patient 05, and △ to patient 06.

Figure 5 summarizes the effect of the NP on tremor amplitude for all the trials in which it was activated, and shows that a consistent reduction was achieved (in 26 out of 30 trials). Overall, tremor amplitude was reduced to a *R*_*att*_=52.33±25.48*%*. The effect of the NP on tremor amplitude was found to be statistically significant (*p*<0.001) when compared to the trials in which it was not activated. The large SD of the overall results originated from the intrinsic variability of the patients’ symptoms, as shown by how widespread the trials performed by the same patients are in the abscissa of Figure 5. Furthermore, more severe tremors were attenuated to a greater extent (*p*=0.008, Mann-Whitney test between the trials with smallest and largest amplitude before the NP was activated). The slope of the linear fit to the attenuation data in Figure 5 (both axes log transformed) was −0.160, which confirmed this trend (*R*^2^=0.243).

**Figure 6:**
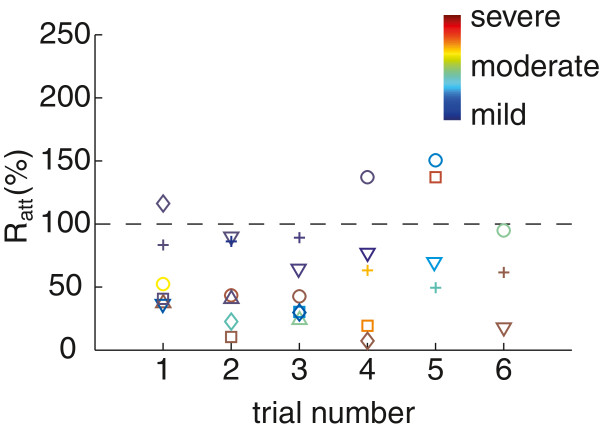
**Tremor attenuation as function of the trial number.** The plot shows how tremor attenuation computed with *R*_*att*_ varied as more trials were performed, for all patients. Each marker represents a single trial, and the color of the marker corresponds to the severity of the tremor during this trial (before the NP was activated). The color scale is adjusted to the amplitude of each patient’s tremor, and represents trials with mild tremor as cold colors, and with severe tremor as hot colors (see colorbar in the plot). Patients are codified as follows: ▿ corresponds to patient 01, □ to patient 02, + to patient 03, ∘ to patient 04, â™¢ to patient 05, and △ to patient 06.

Tremor was exacerbated (*R*_*att*_>100) by the NP in 4 trials, 3 of which corresponded to those in which patients 04 and 05 exhibited a tremor with lower amplitude than observed during calibration (see Figure 6). Given that controller gains were selected manually, and that *K*_*i*_(*tr*_*k*,*k*−1_) was typically chosen to be smaller than *K*_*p*_, we believe that tremor exacerbation could be overcome by improving this method. In the remaining trial, tremor amplitude augmented because the patient’s muscles suffered from accommodation to neurostimulation [47]. Two evidences supported this statement: i) this was the last trial of the session, and in the previous ones the patient exhibited very large tremor attenuation (average reduction of tremor amplitude was *R*_*att*_=26.79±13.13*%*, Figure 5 details all the trials), and ii) the controller applied the maximum current amplitude and did not alleviate the tremor, on the contrary to previous repetitions. However, notice that the performance of the NP was not visibly influenced by trial order, as shown in Figure 6.

**Figure 7:**
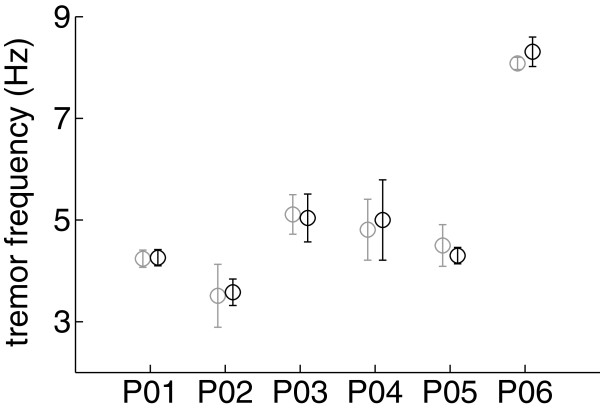
**Effect of the NP on tremor frequency.** The plot compares tremor frequency with the NP activated (black) and not activated (gray) for each patient (denoted as PXX, where XX is the patient code). Results are shown as mean (circle) ± SD (whiskers) of the mean frequency for the ensemble of all trials.

Finally, we analyzed whether tremor attenuation with the NP altered tremor frequency. By comparing tremor frequency with and without the NP activated (for each patient), we observed that it had no effect on tremor frequency (*p*=0.831, see Figure 7), as expected from the central origin of tremor in PD and ET.

### Discussion

These results demonstrate that the NP here presented constitutes a feasible approach to tremor management. Through the control of muscle co-contraction, the NP systematically alleviated the tremor independently from its characteristics, proving that such approach is a viable alternative for treating upper limb tremor. Interestingly, all patients reported that the sensation generated by the NP was tolerable and not unpleasant, and the overall impression was that they could habituate to it. Furthermore, a few patients spontaneously declared that when the NP was activated they could control better their limbs. Users’ perception of the prototype was generally good, and remarkably better than for previous robotic devices.

The NP attained a reduction of tremor amplitude in patients suffering both from PD and ET, in spite of their inherent differences in underlying pathophysiologic mechanisms [4] and symptomatology. Our initial concern was that joint rigidity arising from PD [8] could hinder tremor reduction with the NP in these patients, given that their joints already show increased stiffness and viscosity [53]. The physical reason for this would be a displacement of the natural cut off frequency of their muscles towards lower values (for healthy individuals it is in the range ∼ 2–3 Hz [31,32,34]), which could in turn impede attaining the level of co-contraction necessary to attenuate the tremor. However, our results deemed this hypothesis untrue, even in a patient with severe parkinsonian tremor of low frequency (patient 02, who had a tremor frequency of ∼ 3.5 Hz and experienced a tremor reduction with the NP of *R*_*att*_=26.79±13.13*%*). We believe that the effects of levodopa intake might have facilitated this by decreasing limb rigidity. The fact that the NP successfully alleviated low frequency tremors is of great interest, as the frequency of both essential and parkinsonian tremor experience a decline with time [54], and thus broadens the group of potential users of the system.

In this line, tremor frequency did not to influence the performance of the NP, although our group was biased to patients suffering from low or medium frequency tremor (below ∼ 5 Hz, see Table 1). This characteristic was a consequence of our patient selection because: i) we included two patients suffering from PD, which tremor frequency is typically below 6 Hz [10], and ii) the severity of ET is inversely correlated with its frequency (the slope between the log displacement and the log amplitude is ∼−4 [55]), and our group comprised two subjects with moderate and one with severe tremor (patients 01, 03 and 05). Nevertheless, the performance of the NP for the patient with high frequency tremor (patient 06) was good, achieving an attenuation of *R*_*att*_=34.45±8.87*%*. Although this patient exhibited tremor which amplitude decayed over time (*R*_*att*_=77.35±29.37*%* when the NP was not active), the improvement with the NP was clearly noticeable when both conditions are compared. Furthermore, it was not clear *a priori* whether the NP could attenuate low frequency tremors due to the fact that they manifest close to the natural cut off frequency of muscles, and thus require from a larger alteration of their physiologic response. Given that average attenuation for patient 02 (tremor frequency ∼ 3.5 Hz) was *R*_*att*_=26.79±13.13*%*, and for patient 01 (tremor frequency ∼ 4.2 Hz) was *R*_*att*_=58.99±26.81*%*, we conclude that low frequency tremors can be successfully managed with the NP. These results suggest that tremor frequency is not a criterion that restricts the applicability of the system.

As shown in Figure 7, the NP did not alter tremor frequency in spite of the obvious proprioceptive feedback. This is motivated by the central origin of tremor in PD [8] and ET [4,5], which predominates over the other mechanisms that contribute to their genesis, namely long and short latency reflexes, and mechanical factors [56]. Therefore, our results are in line with evidences on the limited role of sensory feedback in the generation, maintenance and modulation of tremor in PD [8]. ET patients however, exhibit a more evident interaction between the stretch reflex and the tremor itself [22], being the most noticeable example the separation of both components, otherwise entrained, under inertial loading [57]. In this line, visual inspection of Figure 4(F) (bottom plot, the power spectral density) suggests that the central and reflex components were separated in patient 06, both when the NP was activated and not. A more profound investigation of this phenomenon is needed, and would require from the integration of electromyographic (EMG) recordings and advanced signal processing techniques—to remove the artifacts generated by the NP on the EMG signal—in a novel protocol.

Furthermore, given that the NP implements a proportional integral controller, our results suggest that an increase in tremor amplitude was compensated by an increase in the level of muscle co-contraction. As a consequence, we can conjecture that both essential and parkinsonian tremor are reduced when muscle contraction is sufficiently augmented, i.e. when the stiffness and viscosity of the tremulous joint are increased due to their monotonic relationship with the activation level [37]. Comparing this with previous works that assessed the influence of voluntary muscle contraction on the amplitude of tremor, our results are in line with what has been reported for PD [24], but contradicts a recent study that showed how high contraction intensities caused larger amplitude fluctuations in ET [25]. Remarkably, these studies assessed the effect of volitional muscle activation on tremor, while ours deals with artificial activation with a NP, and thus motor control mechanisms are involved in a lesser extent. Therefore, we believe that the pathophysiologic landmarks of PD and ET may account for the difference. Although the cerebellothalamic pathways play a role in the generation of parkinsonian symptoms [58], no evidence of cerebellar involvement apart from a compensative hyperactivation [59]—increased as the disease progresses [60]—, has been found. As for ET, on the contrary, all three cerebellar areas are impaired to a certain extent [7]. Thus we believe that cerebellar disfunction could explain the exacerbation of ET during voluntary contractions, possibly through reticulospinal projections to muscles [61,62].

In a few trials, we observed that tremor migrated towards proximal joints when it was suppressed at the more distal ones; i.e. when it was suppressed at the wrist it appeared or increased at the elbow or at the shoulder. This occurred in patients with both PD and ET, and has been previously reported in two studies dealing with inertial or force loading in tremor. The first was a study on coordination during postural holding in PD, where the authors found that mass loading of the index finger enhanced the tremor at the proximal segments while posture was maintained [63]. The second focused on the attenuation of tremors of different etiology (mainly ET) with a wearable robot that applied a constant viscosity to the affected joints [21]. None of them found a detailed physiologic explanation for tremor migration. Therefore, further research on this topic is needed, given that it constitutes a major aspect when developing NPs or neurorobots for functional compensation of tremor during daily living. Future studies will need to address both the coordination mechanisms *per se*, and the influence that inertial loads and muscle activation have on them.

When compared with current treatments for ET, the reduction of tremor provided by the NP (*R*_*att*_=50.37±29.01*%*) was similar to that of drugs with proved efficacy (∼50*%*[12]), but worse than the results attained with DBS (tremor attenuation ∼ 50 – 80%, [64]). Nevertheless, the latter implies a surgical procedure, while the NP is envisioned as a mock up system that the user may wear underneath his clothes. Furthermore, when compared to pharmacotherapy, the NP reduced tremor amplitude in all 4 ET patients in our group, while drugs are effective in ∼ 50% of patients [65]. No conclusive results can be drawn given the size of our group, although this finding is encouraging. The 2 patients with PD continued their intake of levodopa during the experiments but experienced a reduction of their tremor (*R*_*att*_=42.56±24.91*%*). This suggests that for these patients the NP was an interesting approach to complement pharmacotherapy.

Finally, the limitations of our study need to be acknowledged. First, although our results demonstrate that the NP constitutes a feasible alternative to tremor management, we believe they may be improved by developing an automatic method to select the gains of the controller. This would require the identification of the parameters of a model of muscle response to stimulation, which constitutes our future work. As mentioned above, we believe that better gain selection could have eliminated most trials with tremor exacerbation. Second, for extended use of the NP, it would be necessary to adapt the parameters of the controller when the muscle exhibits accommodation to the ongoing level of neurostimulation. Manual tuning of the parameters by a practitioner, together with adaptive control techniques may provide a solution for this.

## Conclusions

This study presented the design, implementation and validation of a novel NP for tremor management. Experimental results with a representative group of patients (mean reduction of tremor amplitude *R*_*att*_=52.33±25.48*%*, *p*<0.001) proved the concept of tremor attenuation through modulation of muscle co-contraction with an ambulatory NP. Importantly, consistent tremor attenuation was achieved irrespectively from its severity, frequency, and etiology (validation was performed with patients suffering from ET or PD), although there was a trend towards greater reduction of more severe tremors. Importantly, the patients found the sensation induced by the NP not unpleasant, and informed that they could use it during daily living. Furthermore, some of them reported spontaneously that they felt that tremor was reduced when the NP was activated. All patients exhibited a positive response to the NP, which is of great interest given that a significant proportion of those suffering from tremor do not respond to medication (e.g., 50% of ET patients). These results encourage the functional and clinical evaluation of the NP as a non invasive alternative to tremor treatment, either alone, or as a complement to pharmacotherapy.

## Endnote

^a^This rating only included those items related to tremor severity at the most affected limb.

## Abbreviations

ADL: Activity of daily living; DBS: Deep brain stimulator; ET: Essential tremor; EMG: electromyography; NP: Neuroprosthesis; PD: Parkinson’s disease; TVR: Ratio of tremor to voluntary movement.

## Competing interests

The authors declare that they have no competing interests.

## Authors’ contributions

JAG contributed to all the stages of this work (i.e., design of the protocol, implementation of the NP, performance of the experiments, data interpretation and writing). ER and JMBL contributed to the design of the protocol, the performance of the experiments, the interpretation of the data and writing. JLP contributed to the design of the protocol, the interpretation of the data and writing. All authors read and approved the final manuscript.

## Supplementary Material

Additional file 1 **Video that illustrates the performance of the NP.** It shows, for two patients (patients 01 and 02), how tremor amplitude within a trial was remarkably reduced when the NP was activated. Click here for file
